# Nanomedicine strategies for overcoming multidrug resistance in breast cancer: recent advances and future perspectives

**DOI:** 10.1186/s43046-026-00400-8

**Published:** 2026-08-26

**Authors:** Mohit Kumar, Tejaswi, Rohit Bangwal, Manish Yadav, Rahul Singh

**Affiliations:** 1 School of Pharmaceutical Sciences, CGC University, Mohali, Punjab 140301 India; 2 School of Healthcare & Allied Sciences, GD Goenka University, Sohna, Haryana 122103 India; 3 COER University, Roorkee, India

**Keywords:** Multidrug resistance, Breast cancer, Nanomedicine, Nanocarriers, Combination therapy, Cancer stem cells, Targeted drug delivery

## Abstract

Multidrug resistance (MDR) remains a major challenge in the effective treatment of breast cancer, often leading to therapeutic failure, tumor recurrence, and poor clinical outcomes. Multiple mechanisms contribute to MDR, including overexpression of ATP-binding cassette (ABC) transporters, evasion of apoptosis, epithelial–mesenchymal transition (EMT), and the persistence of cancer stem cells (CSCs). In recent years, nanomedicine has emerged as a promising strategy to overcome these resistance pathways by enhancing drug delivery, improving intracellular drug accumulation, and enabling targeted and combination therapies.

This review provides a comprehensive overview of nanomedicine-based approaches for overcoming MDR in breast cancer. Various nanocarrier systems, including liposomes, solid lipid nanoparticles (SLNs), nanostructured lipid carriers (NLCs), polymeric nanoparticles, inorganic nanocarriers, nanoemulsions, and ligand-targeted systems, are systematically discussed in terms of their design, drug delivery capabilities, and role in bypassing resistance mechanisms. Particular emphasis is placed on combination nanotherapeutic strategies, such as co-delivery of chemotherapeutic agents with natural compounds, gene modulators, or sensitizers, which have demonstrated enhanced efficacy in both in vitro and in vivo models.

In addition, this review critically evaluates the limitations of current preclinical studies and highlights key challenges in clinical translation, including variability of the enhanced permeability and retention (EPR) effect, safety concerns, and large-scale manufacturing issues. Overall, nanomedicine offers a multifaceted and promising approach to overcome MDR in breast cancer; however, further translational and clinical studies are required to fully realize its therapeutic potential.

## Introduction

In 2020, 10% of cancer-related fatalities and 13.5% of new cancer cases in India were caused by female breast cancer (BC), which is the country’s top cause of cancer incidence and mortality [1]. Furthermore, from a molecular perspective, BC is a diverse illness. Breast cancer treatment typically involves a combination of surgery, chemotherapy, and radiation therapy.

Furthermore, conventional therapies frequently result in treatment efficacy that is far from satisfactory due to fundamental pharmacokinetic limitations, such as poor drug solubility, systemic toxicity, and the emergence of multidrug resistance (MDR) [2]. The potential of cancer cells to simultaneously develop resistance to many structurally unrelated chemotherapeutic drugs with a unique mode of action and endure therapy is known as MDR [3].

MDR is dependent on a variety of pathways, including changes in the pathways leading to apoptosis, DNA damage response, and its downstream signaling pathways; changes in the proteins responsible for drug transfer from the cell membrane; changes in the enzymes responsible for drug processing and metabolism; changes in the composition of the cell membrane; and changes in the tumor environment, cancer stem cells (CSCs), and epithelial mesenchymal transition [4].

To improve therapeutic outcomes, researchers have increasingly focused on understanding the molecular mechanisms underlying multidrug resistance. Because of its distinct biological and physicochemical properties, nanomedicine—an inventive and alternative application of nanotechnology in medicine—opens up new avenues for reversing MDR. Additionally, chemotherapeutic medications, photosensitizers, immunotherapeutic adjuvants, immune checkpoint inhibitors, and other therapeutic molecules can be delivered to tumor sites by nanomedicine through active, passive, and stimuli-responsive tumor targeting.

This review aims to provide a structured and integrative perspective on nanomedicine-based strategies for overcoming multidrug resistance (MDR) in breast cancer. Unlike previous studies that focus primarily on individual nanocarrier systems or isolated therapeutic approaches, this work systematically links molecular mechanisms of MDR with nanocarrier design principles and functional outcomes. In particular, it emphasizes:

- (i) a comparative evaluation of lipid-based, polymeric, and inorganic nanocarriers in the context of MDR reversal,
- (ii) the role of co-delivery strategies involving chemotherapeutics and sensitizing agents, and
- (iii) emerging targeting approaches addressing cancer stem cells and epithelial–mesenchymal transition.

By integrating mechanistic insights with translational considerations, this review provides a more comprehensive framework for the rational design of next-generation nanotherapeutics.

### Mechanisms of MDR

MDR is caused by a variety of mechanisms. Currently, the well-understood mechanism of MDR is resistance brought on by the overexpression of members of the ATP-binding cassette (ABC) membrane transporter superfamily, which comprises of 48 known genes. MDR-associated protein-1 (MRP1/ABCC1), breast cancer resistance proteins (ABCG2), and P-glycoprotein (P-gp/ABCB1) are some of this superfamily’s best-characterized members. These proteins reduce the effective intracellular concentration of certain endotoxins and chemotherapeutic drugs in an ATP-dependent way. P-glycoprotein (P-gp) is also known as cluster of differentiation 243 (CD243) and multidrug resistance protein-1 (MDR-1). It is also responsible for xenobiotic efflux and is the first member of the ABC superfamily [5, 6]. P-gp overexpression in cancer cells is the cause of MDR.

Several chemotherapeutic drugs bind to the multiple drug-binding sites of P-glycoprotein’s transporter structure [7, 8]. Breast cancer resistance protein (BCRP) was found when two P-gp inhibitors, mitoxantrone and Tariquidar, were given simultaneously to a drug-resistant human breast cancer cell line [9, 10]. The ABCG2 gene encodes BCRP, which belongs to the ABCG subfamily of ABC transporters. BCRP is a half-transporter consisting of an N-terminal NBD and a carboxy-terminal TMD with six membrane-spanning helices [11]. The transporter has a major impact on intercellular drug absorption, metabolism, excretion, and toxicity. It also functions as an efflux pump to move anti-cancer drugs from breast cancer cells to the extracellular environment, giving the tumor cells MDR. Although this transporter was initially identified in breast cancer, it was subsequently discovered to be the cause of MDR in most cancer types [12].

**Fig. 1:**
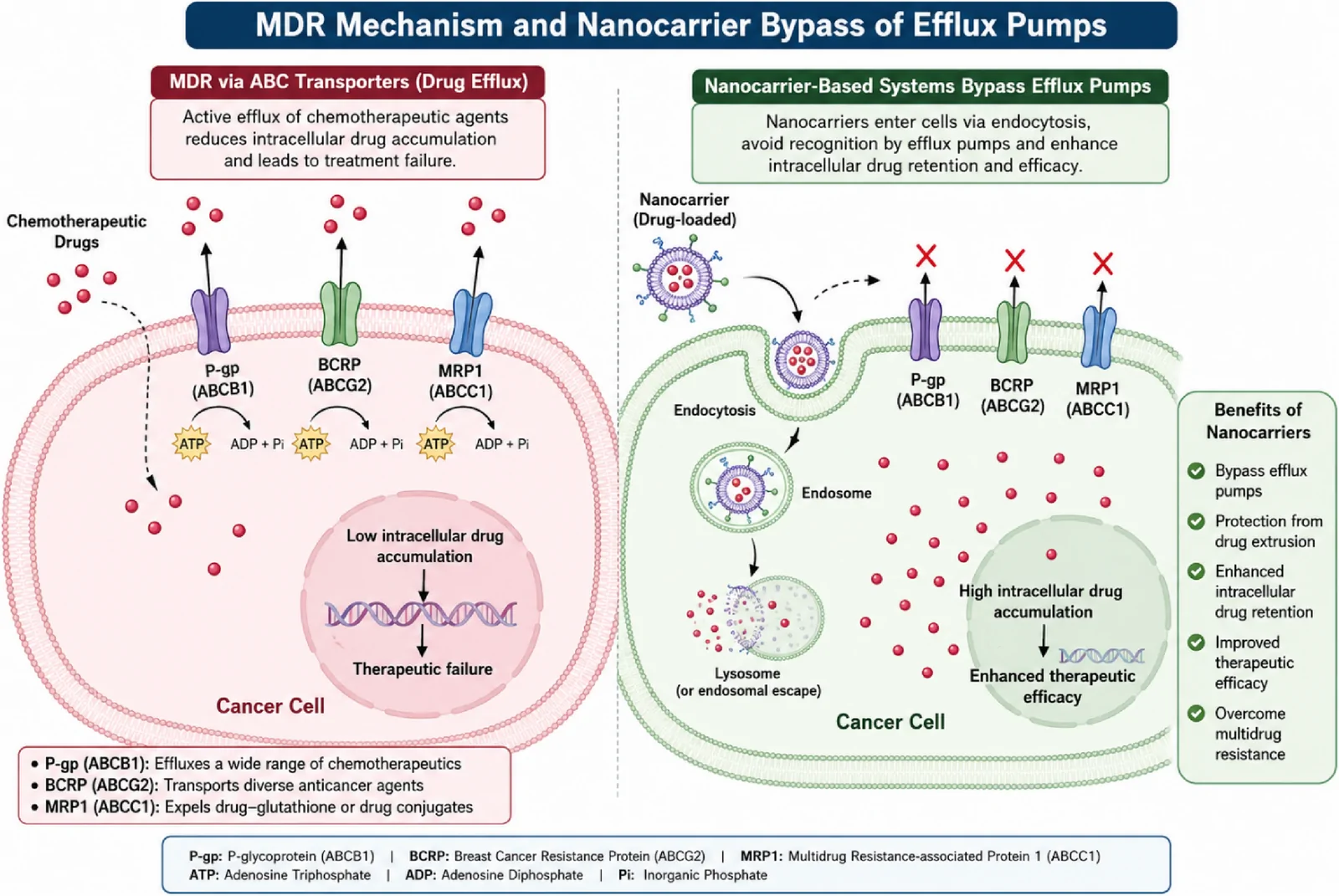
Schematic representation of multidrug resistance (MDR) mediated by ATP-binding cassette (ABC) transporters, including P-glycoprotein (P-gp), BCRP, and MRP1, which actively efflux chemotherapeutic agents out of cancer cells, leading to reduced intracellular drug accumulation and treatment failure. The figure also illustrates how nanocarrier-based systems bypass efflux pumps through endocytosis, resulting in enhanced intracellular drug retention and improved therapeutic efficacy

The human ABC family of cell membrane transporters, which contains the multidrug resistance protein (MRP), is known to be the cause of MDR. This transporter was first identified in the drug-resistant small cell lung cancer cell line H69AR [13]. MRP transports both endogenous and xenobiotic drugs. The role of MRP1 in the emergence of drug resistance in various malignancies has been extensively studied over the last 20 years. This suggests that the MRP1 transport mechanism differs from the P-gp transport mechanism [14]. The majority of human tissues have widespread expression of MRP1. It is hence essential to MDR and present in most cancers, including breast cancer. MRP1 is responsible for resistance to drug families such as vinca alkaloids, anthracyclines, camptothecins, epipodo-phyllotoxins, platinum compounds, nucleoside and nucleotide analogs, folate antimetabolites, and methotrexate drugs. The only taxene to which MRP1 imparts resistance is methotrexate. Recurrence of breast cancer is closely linked to increased MRP1 activity in tumor cells [15]. Anti-cancer medication intracellular accumulation is reduced when MRP/ABCC members (MRPs 1–3) cause resistance, which is frequently brought on by increased efflux. The major mechanisms of ABC transporter-mediated drug efflux and the ability of nanocarrier systems to bypass these resistance pathways through endocytosis are illustrated in Fig. 1.

Beyond classical mechanisms such as drug efflux and apoptosis resistance, multidrug resistance in breast cancer is strongly influenced by dynamic cellular processes like epithelial–mesenchymal transition (EMT) and the presence of cancer stem cells (CSCs). EMT is a biological process in which epithelial cancer cells gradually lose their cell–cell adhesion and polarity, acquiring a more mesenchymal and invasive phenotype. This transition is typically marked by reduced expression of E-cadherin and increased levels of mesenchymal markers such as vimentin and N-cadherin. Functionally, EMT not only promotes metastasis but also makes tumor cells less responsive to chemotherapy by activating survival pathways and enhancing drug efflux capacity [16, 17].

Closely associated with EMT is the emergence of CSCs, a small but highly resilient subpopulation of tumor cells responsible for tumor initiation, progression, and recurrence. In breast cancer, CSCs are commonly identified by markers such as CD44⁺/CD24⁻ and ALDH1 positivity. These cells are inherently resistant to chemotherapy due to several factors, including overexpression of drug transporters, efficient DNA repair mechanisms, and a relatively quiescent state that allows them to evade drug-induced cytotoxicity. As a result, even when the bulk tumor is reduced, CSCs can survive and contribute to relapse and metastasis [18, 19].

Nanomedicine offers a practical way to tackle these interconnected resistance pathways. Unlike free drugs, nanocarriers can enter cells through endocytosis, which helps bypass efflux pumps and improves intracellular drug retention. More importantly, they can be engineered to specifically target CSC populations—for example, using ligands such as hyaluronic acid that bind to CD44 receptors. In addition, nanoplatforms enable the co-delivery of chemotherapeutic agents with gene modulators, such as microRNAs or siRNA, that can suppress EMT-related signaling and help restore a more drug-sensitive epithelial phenotype. By simultaneously targeting EMT processes and CSC populations, nanomedicine-based approaches provide a more comprehensive strategy to overcome resistance and improve therapeutic outcomes in breast cancer [20, 21].

To provide a coherent understanding, the discussion is structured to first outline the molecular and cellular mechanisms underlying multidrug resistance (MDR) in breast cancer, followed by the design principles of nanocarrier systems developed to overcome these barriers. In particular, nanocarrier design strategies are tailored to address both efflux-mediated resistance, such as ATP-binding cassette (ABC) transporter activity, and non-efflux mechanisms, including apoptosis evasion, epithelial–mesenchymal transition, and cancer stem cell–associated resistance. Subsequently, preclinical evidence from in vitro and in vivo studies is discussed, along with key challenges associated with clinical translation.

### Nanomedicine strategies

Over the past few decades, nanomedicine has emerged as a promising approach for addressing multidrug resistance (MDR) in cancer. This field, which integrates nanotechnology with medical science, exploits the unique physicochemical properties of nanoparticles to improve drug delivery and therapeutic outcomes. Nanomaterials, including various nanoparticle-based systems, have shown considerable potential in enhancing the efficacy of anticancer agents by improving biodistribution, enabling controlled drug release, and increasing drug accumulation within resistant tumor cells. As a result, drugs with limited therapeutic indices can be transformed into more effective treatment options when delivered through nanocarrier systems.

Nanomedicine-based strategies have demonstrated significant potential in overcoming MDR in breast cancer by enhancing intracellular drug delivery and bypassing resistance mechanisms. Unless otherwise specified, the findings discussed in this section are derived from a combination of in vitro cell line studies (e.g., MCF-7, MCF-7/ADR, MDA-MB-231) and in vivo animal models (e.g., 4T1 and xenograft mouse models). Various nanocarrier systems have been extensively investigated for their ability to improve drug accumulation, reduce efflux, and enhance therapeutic efficacy in resistant breast cancer models.

### Liposome-based nanoparticles

Liposomes are spherical vesicles composed of phospholipid and cholesterol bilayers that offer two microenvironments for the delivery of hydrophilic and hydrophobic medications [22]. Liposomes are widely used as drug delivery systems because they can encapsulate both hydrophilic and hydrophobic therapeutic agents while improving drug solubility and bioavailability. By increasing nutraceuticals retention and cellular contact, liposomes improve oral bioavailability and prevent GI tract degradation [23]. Polyethylene glycol (PEG) coating liposomes prevents liposome degradation by macrophages in the reticulo-endothelial system (RES) and increases nutraceutical retention [24]. By employing targeting strategies, they take advantage of the enhanced permeability and retention effect (EPR) and binding with overexpressed receptors on the surface of cancer cells. Liposomes increase the concentration of nutraceuticals in cancerous tissues while decreasing their exposure in healthy tissues [25].

Several studies have demonstrated the potential of liposomal formulations to enhance the therapeutic efficacy of anticancer agents such as curcumin, epirubicin, and thymoquinone in breast cancer models. In 2022, Mahrous et al. created liposomes using β-carotene that demonstrated significantly higher cytotoxicity against MCF-7 cells, suggesting improved anticancer potential and bioavailability [26]. Liposomal curcumin nanoparticles as an anticancer agent against the breast cancer cell lines MCF-7 and KHOS, demonstrating significant potential for apoptosis and tumor growth inhibition [27]. In order to treat breast cancer, Odeh et al., (2012) developed liposomal thymoquinone. It successfully stopped the growth of MCF-7 and T47D breast cancer cell lines and showed minimal toxicity in normal fibroblasts [28].

Furthermore, liposomal hesperetin significantly reduced MDA-MB-231 cell sustainability and overcame multidrug resistance proteins [29]. Zhao et al., (2013) created liposomal epirubicin to treat breast cancer. With enhanced cellular permeability and little interference with membrane pump functions, this formulation effectively inhibited the growth of cancer cells in MDA-MB-435 and its resistant variant, MDA-MB-435ADR [30]. In contrast to free cisplatin alone, liposomal nanoparticles co-loaded with cisplatin and curcumin dramatically reduced breast cancer cell viability by 82.5%, exhibiting a synergistic anticancer effect [31]. Liposomes containing doxorubicin, pachymic acid, and dehydrotumulosic acid improved anticancer activity in MDA-MB-231 cell [32], and formulations that co-encapsulated doxorubicin and berberine significantly slowed tumor growth and reversed doxorubicin-induced cardiac toxicity in 4T1 Model [33].

Despite these encouraging findings, most liposomal studies are limited to in vitro breast cancer cell lines such as MCF-7 and MDA-MB-231, with relatively few validated in vivo or clinical studies, and issues such as drug leakage and variability in the EPR effect may affect their translational reliability.

### Phytosome-based nanoparticles

Phytosomes are nanocarriers formed by complexing phytochemicals with phospholipids, which enhances their stability and bioavailability [34]. Although they share structural similarities with liposomes, their distinct structural makeup makes them more effective at delivering specific nutraceuticals to cancer sites. The phytosomes’ phospholipid bilayer facilitates contact-facilitated drug transport, which allows nutraceuticals to diffuse into cancer cells through a lipid-lipid interaction between the cell membrane and carrier. Additionally, phytosomes can enhance the diffusion of lipophilic nutraceuticals through the small intestinal brush barrier. The controlled and prolonged release of nutraceuticals into the systemic circulation is typically hampered by hydrolytic digestion and the agglomeration of these agents. To stop drug agglomeration, phytosomes’ phosphatidylcholine forms a monolayer in the GIT [35]. Phytosomes therefore increase the bioavailability of nutraceuticals, enhancing their effectiveness and permitting non-invasive delivery [36].

Alhakamy et al. created quercetin-loaded phytosomes for MCF-7 breast cancer cells. The phytosomal formulation decreased inflammatory markers (NF-kB, TNF-α), induced apoptosis, and enhanced cellular sensitivity to quercetin [37]. Sabzichi et al. created phytosomal luteolin that targets MDA-MB-231 cells, resulting in significantly higher cancer cell mortality through the suppression of Nrf2 gene expression [38]. Aloe vera extract was prepared in phytosomes by Murugesan et al., demonstrating efficient cytotoxicity against MCF-7 cells with better biocompatibility than unloaded extract [39].

Although phytosomes show improved bioavailability and promising activity in cell-based studies, their application remains largely restricted to phytochemicals, with limited in vivo validation and insufficient evidence regarding long-term stability and clinical translation.

### Solid lipid nanoparticles (SLNs)

SLNs, which are used to encapsulate drugs, are made of solid lipid components combined with surfactants. They range in size from 50 to 500 nm [40]. The solid lipid matrix of SLNs enables controlled drug release due to its zero-order degradation kinetics. SLNs’ solid lipid core shields nutraceuticals from chemical deterioration and permits sustained release [41].

Rahman et al. created silymarin-loaded SLNs to treat breast cancer. Compared to plain silymarin, the SLNs significantly reduced MCF-7 cell proliferation [42]. Hatami et al. developed quercetin SLNs for triple-negative breast cancer. In comparison to unencapsulated quercetin, these reduced angiogenesis and colony formation, enhanced apoptosis through Bcl-2/Bax gene regulation, and targeted MCF-7 and MDA-MB-231 cells [43]. Beta-carotene-loaded SLNs showed 1.92 times greater bioavailability and enhanced anticancer activity against MCF-7 breast cancer cells [44]. EGCG-loaded SLNs demonstrated 8.1 times greater cytotoxicity than free EGCG against MDA-MB-231 breast cancer cells [45]. Specifically for SKBR3 breast cancer cells, curcumin-loaded SLNs were created with elevated cytotoxicity and apoptotic rate [46].

However, despite their stability and sustained release properties, SLNs are often limited by drug expulsion during storage and a lack of consistent in vivo validation, which may affect their reliability in long-term MDR management.

### Nanostructured lipid carriers (NLCs)

NLCs have demonstrated their capacity as sophisticated carriers to overcome the previously mentioned limitations of SLNs [47]. These have flaws in the lipid matrix because they are made up of both liquid and solid lipids. Larger amounts of nutraceuticals can be incorporated into NLCs due to the lipid matrix’s imperfections, which also reduce expulsion during polymorphic transitions and increase the gap between fatty acid chains [48, 49]. Nutraceuticals can be targeted through the lymphatic system thanks to NLCs, which has several advantages, including avoiding hepatic metabolism, lowering hepatotoxicity, and increasing bioavailability [50]. Furthermore, NLCs offer all of the advantages of SLNs.

Chitosan-coated NLCs loaded with tetrahydrocurcumin improved skin penetration, cytotoxicity, and cell uptake for MDA-MB-231 breast cancer cells [51]. Mangla et al. created NLCs co-loaded with sulforaphane and tamoxifen, which decreased the toxicity of tamoxifen while increasing the oral bioavailability of sulforaphane by 4.8 times and tamoxifen by 5.2 times [52]. Morin and quercetin were combined to create NLCs, which demonstrated noticeably greater cytotoxicity against MCF-7 breast cancer cells [53]. NLCs loaded with doxorubicin, docosahexaenoic acid, and α-tocopherol succinate showed improved anticancer activity and decreased toxicity in 4T1 breast cancer [54]. Alhalmi et al. created NLCs co-loaded with naringenin and raloxifene, which showed better antitumor and antioxidant activity than traditional suspensions [55].

While NLCs demonstrate improved drug loading and stability compared to SLNs, most studies remain at the preclinical stage, and further investigation is required to assess their large-scale manufacturability and clinical applicability.

### Inorganic nanocarriers (Gold, magnetic, quantum dots)

Silver, gold, silica, iron oxide, and other inorganic materials make up the core of inorganic nanocarriers, while organic materials like polymers make up the shell. The shell protects the confined molecules from external physiological changes and acts as a site of attachment for biomolecules or receptors [56]. These nanocarriers can be employed as therapeutic agents and drug carriers due to their properties, which include photosensitivity, conductivity, magnetic attraction, and thermal stability. Anticancer molecules can be encapsulated with a synergistic effect thanks to the therapeutic potential of inorganic nanocarriers [57]. Metals, metal oxides, and non-metallic materials (silica and carbon) can all be used to create INPs. They offer a number of advantages as drug carriers, including enhanced drug loading capacity and the ability to participate in photothermal therapy (PTT) and photodynamic therapy (PDT) [58]. Numerous characteristics, such as distinct physiochemical characteristics (size, shape), increased surface area, chemical composition, and proficiency to functionalization for improved targeting at the tumor site, make these potentially useful carriers for nutraceuticals [59]. It has been claimed that gold nanoparticles (AuNPs) have better targeting, gene silencing, and drug delivery capabilities, particularly when they have ligands that allow for controlled deposition into cancer cells [60].

AuNPs loaded with curcumin exhibited enhanced apoptotic and antiproliferative activity against MCF-7 breast cancer cells [61]. AuNPs of quercetin reduced the viability of capillary-like structures in HUVECs and more effectively prevented the migration and invasion of MCF-7 and MDA-MB-231 breast cancer cells than free quercetin [62]. Curcumin-loaded quantum dots demonstrated substantial selective cytotoxicity, with no effect on normal HEK-293 cells, but only approximately 10% cell viability in MCF-7 breast cancer cells [63].

Despite their multifunctional properties and strong therapeutic potential, concerns regarding long-term toxicity, biodistribution, and accumulation in non-target tissues remain significant challenges for their clinical translation.

### Nanoemulsions (NEs)

Nanoemulsions are kinetically stable but thermodynamically unstable colloidal dispersions that range in size from 20 to 200 nm [64]. They engage in unique bio-nano interactions, allow for extended circulation, and deeply penetrate tissues due to their nanoscale size [65]. Because of their ability to contain nutraceuticals within food matrices, preserve their advantageous qualities during storage, and permit their controlled release through the gastrointestinal tract, NEs are efficient means of encapsulating, transporting, and delivering nutraceuticals [66]. By offering a larger surface area for better interaction at the target biological locations, NEs significantly decreased the risk of nutraceuticals degrading during the digestive process, resulting in improved bioabsorption [67]. They can encapsulate both hydrophilic and lipophilic nutraceuticals, boosting their in vitro activity and bioavailability [68]. They come in two varieties: water-in-oil (W/O) and oil-in-water (O/W). NEs have been successfully used as nanocarriers to deliver a variety of nutraceuticals.

For 4T1 breast cancer, NEs that combined paclitaxel and baicalein resulted in better drug accumulation at the tumor site and improved bioavailability by inducing cellular ROS generation, cell cycle arrest, and enhanced mitochondrial membrane potential suppression [69]. Ceramella et al. created nanoemulsions that co-encapsulated quercetin and cisplatin and demonstrated synergistic cytotoxicity, particularly against drug-resistant MDA-MB-231 breast cancer cells [70].

Although nanoemulsions show enhanced bioavailability and promising in vitro efficacy, their thermodynamic instability and limited long-term in vivo validation may restrict their widespread application in MDR treatment.

### Polymeric nanoparticles (PNPs)

The therapeutic agents can be encapsulated, physically adsorbed on the carrier’s surface, or chemically bonded to the surface of PNPs, which are solid colloidal particles with a size range of 10–1000 nm and are composed of biodegradable polymers [71, 72]. These offer efficient delivery at the intended locations and enhanced safety for encapsulated nutraceuticals. Furthermore, PNPs have a number of beneficial properties, including their nanoscale (less than 200 nm), higher surface volume ratio, biocompatibility, and susceptibility to surface modification [73, 74]. Nanoparticles that are smaller than 200 nm have an enhanced permeability and retention (EPR) effect, which increases their penetration and prolongs their retention in the tumor environment [58]. Anticancer agents are able to target specific tumor locations because they have multiple functional groups on their surface [75]. PNPs can be designed to withstand phagocyte engulfment, which enhances cellular absorption and prolongs circulation time, resulting in increased efficiency.

For SK-Br-3 breast cancer, PNPs co-loaded with curcumin and methotrexate had a much lower IC50 and synergistically enhanced cytotoxicity [76]. PNPs loaded with naringenin and doxorubicin for the breast cancer cell lines MDA-MB-231, MCF-7, T47D, and HBL-100 demonstrated effective cell uptake and tumor growth inhibition, lowering tumor volume while preserving stable bodyweight [77]. For 4T1 and MDA-MB-231 breast cancer, curcumin and paclitaxel co-loaded nanoparticles produced better cancer suppression and therapeutic results than single-agent formulations [78].

Despite their versatility and strong potential for co-delivery strategies, polymeric nanoparticles often involve complex synthesis processes and may present biocompatibility and scalability challenges that need to be addressed for clinical use.

### Ligand-targeted nanoformulations

The challenges of targeting cancer cells by overcoming biological barriers, off-target effects, and biodistribution problems limit the clinical applicability of nutraceutical nanoformulations in the treatment of cancer [79]. Therefore, it is necessary to design selective nanocarriers that can target particular tumor cells. Ligand-targeted nanoparticles are designed by conjugating nanocarriers with molecules such as antibodies, peptides, or nucleic acids. These ligands recognize receptors that are overexpressed on cancer cells, enabling selective drug delivery [80, 81].

In 2020, Kazi et al. synthesized folate-targeted PLGA nanoparticles and demonstrated improved cytotoxicity and cellular uptake with EGCG against MDA-MB-231 breast cancer cells [82]. Folate-functionalized nanoparticles with EGCG increased selective toxicity toward cancer cells while sparing normal cells [83]. Folate receptor-targeted nanoparticles encapsulating luteolin achieved three times greater tumor suppression in 4T1 cells breast cancer model [84]. Meng et al. created folate-conjugated albumin nanoparticles loaded with baicalin, which significantly increased tumor suppression, enhanced uptake, and induced apoptosis in comparison to plain baicalin in MCF-7 [85].

While ligand-targeted systems offer improved specificity and cellular uptake, their effectiveness may be influenced by factors such as receptor heterogeneity, protein corona formation, and variability in targeting efficiency in vivo.

### Combination nanomedicine strategies for overcoming MDR in breast cancer

Recent advances in nanomedicine have increasingly emphasized the importance of combination therapeutic strategies to overcome multidrug resistance (MDR) in breast cancer by simultaneously targeting multiple resistance pathways. Given the complexity of MDR mechanisms—including drug efflux, apoptosis evasion, tumor microenvironment adaptation, and cancer stem cell survival—monotherapies often fail to achieve durable responses. In this context, multifunctional nanocarriers capable of co-delivering chemotherapeutic agents with other therapeutic modalities have emerged as a highly promising approach to enhance treatment efficacy and reverse resistance.

Among these, chemotherapy combined with gene therapy has demonstrated significant potential in suppressing resistance-associated genes such as MDR1 and ABCB1. Nanoparticle-mediated co-delivery of doxorubicin and siRNA targeting P-glycoprotein has been shown to effectively downregulate efflux transporters and enhance intracellular drug accumulation in resistant breast cancer cells. For instance, Yan et al. (2023) reported that siRNA-loaded nanocarriers significantly improved chemosensitivity by silencing MDR-related genes [86], while Zeng et al. (2023) highlighted the role of nano-delivery systems in restoring drug efficacy in triple-negative breast cancer models through gene modulation [87]. These findings demonstrate that gene–drug co-delivery systems can directly target the molecular basis of MDR.

Similarly, the integration of chemotherapy with photothermal therapy (PTT) has shown promising results in overcoming drug resistance. Photothermal nanomaterials, such as metal and metal oxide nanoparticles, can generate localized hyperthermia upon near-infrared (NIR) irradiation, leading to enhanced membrane permeability, disruption of tumor cell integrity, and inhibition of ATP-dependent drug efflux pumps. Subhan (2022) demonstrated that metal oxide nanoparticle-based systems significantly improved drug accumulation and cytotoxicity in MDR breast cancer models [88]. In parallel, Gote et al. (2021) reported that tumor-targeted nanomedicine platforms utilizing photothermal effects enhanced therapeutic outcomes by facilitating intracellular drug delivery and overcoming resistance mechanisms [89].

Photodynamic therapy (PDT), when combined with chemotherapy, represents another effective strategy for MDR reversal. PDT-based nanoplatforms generate reactive oxygen species (ROS), which induce oxidative stress, mitochondrial dysfunction, and subsequent cancer cell death. Importantly, ROS generation can impair ATP production, thereby reducing the activity of drug efflux transporters. Panigrahi et al. (2024) demonstrated that nanoparticle-mediated PDT significantly enhanced cytotoxicity in breast cancer models through ROS-induced apoptosis [90], while Subhan (2022) further reported that metal oxide-based systems effectively improved therapeutic outcomes by combining oxidative damage with drug delivery. These findings highlight the ability of chemo-PDT systems to bypass conventional resistance pathways [88].

The combination of chemotherapy with immunotherapy has also gained considerable attention, particularly in the context of triple-negative breast cancer. Nanocarriers can be engineered to co-deliver chemotherapeutic agents alongside immune checkpoint inhibitors or immunomodulatory molecules, thereby enhancing both direct tumor killing and immune-mediated responses. Zeng et al. (2023) demonstrated that nanoparticle-based delivery systems could improve immune activation and therapeutic efficacy in resistant breast cancer models [87]. Furthermore, nanomedicine platforms capable of inducing immunogenic cell death have been shown to enhance antigen presentation and overcome immune evasion, representing a critical advancement in MDR-targeted therapy.

In addition to synthetic agents, natural compounds such as curcumin and quercetin have been widely explored as MDR modulators due to their ability to inhibit NF-κB signaling, suppress ABC transporters, and promote apoptosis. Nanocarrier-based co-delivery of these phytochemicals with chemotherapeutic drugs has demonstrated synergistic effects, improving drug bioavailability and reversing resistance. Gote et al. (2021) reported that phytochemical-loaded nanoparticles enhanced drug sensitivity and reduced tumor progression [89], while Velapure et al. (2024) highlighted the role of tumor microenvironment-responsive nanoformulations in improving therapeutic outcomes in breast cancer [91]. These studies support the integration of natural agents into combination nanomedicine strategies.

Targeting autophagy represents another promising approach for overcoming MDR, as autophagy often functions as a cytoprotective mechanism in resistant tumors. Nanoparticles co-loaded with chemotherapeutic agents and autophagy inhibitors have been shown to block survival pathways and enhance apoptosis. Velapure et al. (2024) demonstrated that modulation of the tumor microenvironment through nanotechnology could significantly improve therapeutic efficacy [91], while Gote et al. (2021) emphasized the importance of inhibiting autophagy to sensitize resistant cancer cells [89]. These findings suggest that autophagy-targeted nanotherapy can effectively enhance the response to chemotherapy.

More recently, ferroptosis—a regulated, iron-dependent form of cell death characterized by lipid peroxidation—has emerged as a novel strategy to eliminate MDR cancer cells. Unlike apoptosis, ferroptosis bypasses traditional resistance pathways, making it particularly effective against drug-resistant tumors. Gao et al. (2025) reported that nanomaterial-based ferroptosis induction significantly enhanced anticancer efficacy in breast cancer models [92], while Subhan (2022) demonstrated that iron-based nanoparticles could promote oxidative stress and improve therapeutic outcomes [88]. These findings indicate that ferroptosis-inducing nanoplatforms represent a promising frontier in MDR-targeted therapy.

Collectively, these combination nanomedicine strategies provide a comprehensive and multifunctional approach to overcoming MDR in breast cancer by integrating multiple therapeutic mechanisms within a single platform. By simultaneously modulating drug efflux, enhancing intracellular delivery, inducing alternative cell death pathways, and targeting the tumor microenvironment, these systems offer significant advantages over conventional therapies. However, further research is required to address challenges related to clinical translation, long-term safety, and large-scale manufacturing before these advanced nanotherapeutic systems can be fully implemented in clinical practice.

### Comparative evaluation of nanocarriers

A clear comparison of different nanocarrier systems is essential to understand how effectively they can overcome multidrug resistance (MDR) in breast cancer (Table 1). While most nanocarriers are designed to bypass ATP-binding cassette (ABC) transporter-mediated drug efflux—mainly by promoting cellular uptake through endocytosis rather than passive diffusion—their actual performance in reversing MDR depends on their structure, drug-loading ability, stability, and targeting potential.

**Table 1:** Comparative evaluation of nanocarriers for overcoming multidrug resistance in breast cancer

Nanocarrier Type	Drug Loading Capacity	Ability to Bypass MDR (*P*-gp)	Release Profile	Suitability for Combination Therapy	Advantages	Limitations	Clinical Status
Liposomes	High (hydrophilic + lipophilic)	Moderate–High (via endocytosis)	Controlled	High	Biocompatible, clinically approved, dual loading	Drug leakage, MPS clearance	Clinically established
Phytosomes	Moderate (phytochemicals)	Moderate (via pathway modulation)	Moderate	Moderate	Improved bioavailability of natural compounds	Limited drug versatility	Preclinical/limited clinical
SLNs	Moderate	Moderate	Sustained	Moderate	Good stability, protects drugs	Drug expulsion, low loading	Preclinical
NLCs	High	High	Sustained & improved	Very High	High loading, better stability, co-delivery	Complex formulation	Preclinical/early clinical
Polymeric NPs	Very High	High	Tunable	Excellent	Controlled release, gene + drug delivery	Potential toxicity, complex synthesis	Preclinical/clinical trials
Inorganic NPs	High	Moderate–High	Variable	High (PTT/PDT)	Imaging + therapy, strong stability	Long-term toxicity concerns	Mostly preclinical

Liposomes are the most widely studied and clinically established nanocarriers. One of their key advantages is their ability to carry both hydrophilic and lipophilic drugs, which makes them particularly useful for combination therapies involving chemotherapeutic agents and MDR modulators. They can partially overcome P-glycoprotein (P-gp)-mediated drug efflux by facilitating intracellular delivery through endocytosis. However, liposomes can be rapidly cleared from circulation by the mononuclear phagocyte system (MPS) and may suffer from drug leakage unless modified, for example, by PEGylation to improve their stability and circulation time.

Phytosomes, on the other hand, are specifically designed to enhance the bioavailability of phytochemicals such as curcumin and quercetin through phospholipid complexation. These systems are particularly useful when natural compounds are used as chemosensitizers to inhibit MDR pathways. However, their application is relatively limited because they are mainly suitable for phytoconstituents and offer less flexibility in drug loading compared to other nanocarriers.

Solid lipid nanoparticles (SLNs) provide better physical stability and controlled drug release due to their solid lipid core. This helps protect drugs from degradation and allows for sustained release, which may improve drug retention in resistant tumor cells. However, their highly ordered structure can sometimes lead to drug expulsion during storage, which may reduce their long-term effectiveness.

Nanostructured lipid carriers (NLCs) were developed to overcome this limitation of SLNs. By combining solid and liquid lipids, they form a less ordered structure with more space for drug incorporation. This allows higher drug loading and reduces the risk of drug expulsion. As a result, NLCs are often more efficient for co-delivery strategies, helping to maintain higher intracellular drug levels and improve the reversal of P-gp-mediated resistance. They also tend to show better stability and pharmacokinetic behavior compared to SLNs.

Overall, each nanocarrier system offers specific advantages, but their effectiveness in MDR reversal varies depending on the therapeutic goal. Liposomes are well-established for clinical use, while NLCs offer improved loading and stability. Therefore, selecting an appropriate nanocarrier should be based on its ability to enhance intracellular drug delivery, bypass resistance mechanisms, and maintain a favorable safety and pharmacokinetic profile. A comparative summary of these features is provided in Table 1.

### Challenges and limitations

Despite the profound and well-documented therapeutic potential of nanomedicine in reversing MDR, the transition from optimized laboratory formulations to universally effective clinical treatments is hindered by severe physiological and manufacturing limitations.

A primary and deeply persistent biological challenge is the spontaneous formation of the protein corona. The moment a meticulously engineered nanocarrier enters the complex human bloodstream, hundreds of ubiquitous serum proteins aggressively bind to its external surface. This rapid opsonization inherently alters the precise hydrodynamic size, surface charge, and meticulously engineered biological identity of the particle. More critically, the dense protein corona can entirely obscure conjugated active targeting ligands—such as folate or engineered antibodies—directly preventing the necessary receptor recognition required for targeted drug delivery. Consequently, active targeting mechanisms that perform flawlessly in protein-free in vitro assays frequently become therapeutically useless in vivo.

Manufacturing scalability and strict batch-to-batch reproducibility present an equally formidable industrial obstacle. The rigorous synthesis of multi-component, ligand-targeted nanocarriers, particularly complex NLCs and functionalized polymeric architectures, often requires highly controlled microfluidic channels or sophisticated ultrasonication techniques. Transitioning these precise laboratory-scale procedures to massive, industrial-scale GMP (Good Manufacturing Practice) manufacturing while strictly maintaining exact nanoparticle size distributions, precise drug encapsulation efficiency, and uniform ligand density is technically complex and financially demanding. Furthermore, standard industrial sterilization techniques, such as intense autoclaving or gamma irradiation, frequently disrupt the fragile lipid bilayers of liposomes or fundamentally alter the phase-stability of kinetically stable nanoemulsions, presenting a severe regulatory and logistical bottleneck for commercialization.

Long-term nanotoxicity, particularly concerning inorganic structures like gold nanoparticles and quantum dots, also remains heavily scrutinized by global regulatory bodies. While the organic shells and robust biodegradable properties of SLNs and PNPs offer a high degree of biocompatibility, allowing the body to naturally metabolize the carriers into harmless byproducts, the heavy metal cores of inorganic nanoparticles are uniquely prone to prolonged biological accumulation. Because they cannot be rapidly enzymatically degraded, these rigid inorganic cores often sequester in vital filtration organs such as the liver and spleen for extended periods, raising profound concerns regarding chronic cellular toxicity and sustained oxidative stress.

### Clinical translation

Despite the promising results reported in preclinical studies, the clinical translation of nanomedicine-based strategies for overcoming multidrug resistance (MDR) in breast cancer remains limited. One of the major challenges is the variability of the enhanced permeability and retention (EPR) effect in human tumors, which is often less consistent and less pronounced than in commonly used animal models. This variability can significantly affect nanoparticle accumulation and therapeutic efficacy. In addition, long-term safety concerns, particularly for inorganic nanomaterials, including potential accumulation and chronic toxicity, have not yet been fully addressed. Practical challenges such as large-scale manufacturing, batch-to-batch reproducibility, and regulatory approval further complicate clinical development. Moreover, there is still a lack of well-designed clinical trials specifically targeting MDR reversal, highlighting the need for stronger integration between preclinical findings and clinical validation. Addressing these challenges will be essential for translating nanomedicine-based approaches into effective clinical therapies.

To provide a clearer comparative perspective, the major nanomedicine-based strategies investigated for overcoming multidrug resistance (MDR) in breast cancer are summarized in Table 2. The table integrates data across different nanocarrier systems, therapeutic combinations, and experimental models, enabling direct comparison of their efficacy and underlying mechanisms. Importantly, it highlights the growing role of combination nanotherapies in enhancing drug accumulation, reducing efflux, and improving overall therapeutic outcomes in resistant breast cancer.

**Table 2:** Nanomedicine-based strategies for overcoming MDR in breast cancer

Nanocarrier Type	Drug(s) / Payload	Model Used	Key Outcome	Mechanism / Remark	Ref.
Liposomes	β-carotene	MCF-7 cells	Increased cytotoxicity	Improved bioavailability	26
Liposomes	Curcumin	MCF-7, KHOS cells	Apoptosis, tumor inhibition	Anti-proliferative	27
Liposomes	Thymoquinone	MCF-7, T47D cells	Growth inhibition, low toxicity	Selective anticancer effect	28
Liposomes	Hesperetin	MDA-MB-231 cells	Reduced viability	MDR protein inhibition	29
Liposomes	Epirubicin	MDA-MB-435, MDA-MB-435ADR	Tumor inhibition	Bypass P-gp efflux	30
Liposomes	Cisplatin + Curcumin	Breast cancer cells	82.5% viability reduction	Synergistic effect	31
Liposomes	DOX + Pachymic acid + Dehydrotumulosic acid	In vitro	Enhanced anticancer activity	Multi-drug synergy	32
Liposomes	DOX + Berberine	Tumor model	Reduced tumor growth	MDR reversal, cardioprotection	33
Phytosomes	Quercetin	MCF-7 cells	↓ NF-kB, TNF-α, apoptosis	Anti-inflammatory sensitization	37
Phytosomes	Luteolin	MDA-MB-231 cells	Increased cell death	Nrf2 suppression	38
Phytosomes	Aloe vera extract	MCF-7 cells	Cytotoxicity	Enhanced delivery	39
SLNs	Silymarin	MCF-7 cells	Reduced proliferation	Sustained release	42
SLNs	Quercetin	MCF-7, MDA-MB-231	Anti-angiogenic, apoptosis	Bcl-2/Bax regulation	43
SLNs	β-carotene	MCF-7 cells	1.92× bioavailability	Improved delivery	44
SLNs	EGCG	MDA-MB-231 cells	8.1× cytotoxicity	Enhanced uptake	45
SLNs	Curcumin	SKBR3 cells	Increased apoptosis	Targeted cytotoxicity	46
NLCs	Tetrahydrocurcumin	MDA-MB-231 cells	↑ uptake, cytotoxicity	Chitosan-coated targeting	51
NLCs	Sulforaphane + Tamoxifen	Not specified	↑ bioavailability	Reduced toxicity	52
NLCs	Morin + Quercetin	MCF-7 cells	Higher cytotoxicity	Synergistic	53
NLCs	DOX + DHA + α-tocopherol	4T1 model	Improved activity	Combination therapy	54
NLCs	Naringenin + Raloxifene	Tumor model	Enhanced antitumor activity	Antioxidant + anticancer	55
Inorganic NPs	Curcumin-AuNPs	MCF-7 cells	Apoptosis	Targeted delivery	61
Inorganic NPs	Quercetin-AuNPs	MCF-7, MDA-MB-231, HUVEC	↓ migration, angiogenesis	Anti-metastatic	62
Inorganic NPs	Curcumin-QDs	MCF-7 cells	Selective cytotoxicity	Minimal normal toxicity	63
Nanoemulsions	Paclitaxel + Baicalein	4T1 model	↑ accumulation, ROS	Synergistic effect	69
Nanoemulsions	Quercetin + Cisplatin	MDA-MB-231 cells	Synergistic cytotoxicity	MDR targeting	70
Polymeric NPs	Curcumin + Methotrexate	SK-Br-3 cells	↓ IC50	Synergistic cytotoxicity	76
Polymeric NPs	Naringenin + DOX	MDA-MB-231, MCF-7, T47D, HBL-100	Tumor inhibition	Improved uptake	77
Polymeric NPs	Curcumin + Paclitaxel	4T1, MDA-MB-231	Better suppression	Combination therapy	78
Targeted NPs	EGCG (Folate-PLGA)	MDA-MB-231 cells	↑ uptake, cytotoxicity	Folate targeting	82
Targeted NPs	EGCG (Folate NP)	Cancer vs. normal cells	Selective toxicity	Target specificity	83
Targeted NPs	Luteolin (Folate NP)	Tumor model	3× tumor suppression	Active targeting	84
Targeted NPs	Baicalin (Albumin-folate NP)	Tumor model	↑ apoptosis	Enhanced delivery	85

### Future perspectives

Future research should focus on improving the clinical translation of nanomedicine-based therapies by addressing key challenges such as regulatory approval, large-scale manufacturing, and reproducibility. In particular, there is growing interest in the development of stimuli-responsive nanocarriers that can selectively release therapeutic agents in response to tumor-specific conditions, such as acidic pH, hypoxia, or enzyme overexpression, thereby improving targeting precision and minimizing off-target effects. Additionally, targeting cancer stem cells (CSCs), which play a central role in drug resistance and tumor recurrence, represents a critical direction for future investigation. Designing nanocarriers that can specifically recognize CSC-associated markers, such as CD44, or modulate epithelial–mesenchymal transition pathways may significantly improve long-term therapeutic outcomes.

Furthermore, the advancement of dual-loading and combination strategies is expected to play a pivotal role in overcoming multidrug resistance. Co-delivery systems that integrate conventional chemotherapeutic agents with natural compounds, such as doxorubicin combined with berberine or curcumin, have shown promising potential in enhancing therapeutic efficacy while reducing systemic toxicity, including cardiotoxic effects. Moving forward, the integration of these approaches with personalized and targeted nanomedicine strategies may further improve treatment outcomes and facilitate successful clinical translation.

## Conclusion

Multidrug resistance continues to be a significant barrier in the successful treatment of breast cancer, driven by complex and interconnected biological mechanisms such as drug efflux, apoptosis resistance, EMT, and cancer stem cell–mediated survival. Conventional therapies often fail to address these multifactorial resistance pathways, highlighting the need for more advanced and targeted treatment strategies.

Nanomedicine has shown considerable promise in overcoming these limitations by enhancing drug delivery, improving intracellular retention, and enabling targeted and combination therapies. Various nanocarrier systems, including liposomes, SLNs, NLCs, polymeric nanoparticles, inorganic nanocarriers, and ligand-targeted formulations, have demonstrated the ability to bypass efflux mechanisms and improve therapeutic outcomes in resistant breast cancer models. In particular, combination nanomedicine strategies have emerged as a powerful approach, allowing simultaneous modulation of multiple resistance pathways and resulting in improved efficacy compared to single-agent therapies.

Despite these encouraging findings, most studies remain at the preclinical stage, and several challenges hinder clinical translation. These include variability in nanoparticle accumulation in human tumors, long-term safety concerns, and difficulties in large-scale production and regulatory approval. Therefore, future research should focus on bridging the gap between preclinical success and clinical application through well-designed translational studies and clinical trials.

## Data Availability

No datasets were generated or analysed during the current study.
